# AR-12 Exhibits Direct and Host-Targeted Antibacterial Activity toward *Mycobacterium abscessus*

**DOI:** 10.1128/AAC.00236-20

**Published:** 2020-07-22

**Authors:** Shaoyan Zhang, Yuzhen Zou, Qi Guo, Jianhui Chen, Liyun Xu, Xiaoyu Wan, Zhemin Zhang, Bing Li, Haiqing Chu

**Affiliations:** a Department of Respiratory Medicine, Shanghai Pulmonary Hospital, Tongji University School of Medicine, Shanghai, China; b Tongji University School of Medicine, Shanghai, China; c Shanghai Key Laboratory of Tuberculosis, Shanghai Pulmonary Hospital, Tongji University School of Medicine, Shanghai, China

**Keywords:** *Mycobacterium abscessus*, AR-12 (OSU-03012), intracellular, mouse model

## Abstract

Therapeutic options for Mycobacterium abscessus infections are extremely limited. New or repurposed drugs are needed. The anti-M. abscessus activity of AR-12 (OSU-03012), reported to express broad-spectrum antimicrobial effects, was investigated *in vitro* and *in vivo*. Antimicrobial susceptibility testing was performed on 194 clinical isolates. Minimum bactericidal concentration and time-kill kinetics assays were conducted to distinguish the bactericidal versus bacteriostatic activity of AR-12. Synergy between AR-12 and five clinically important antibiotics was determined using a checkerboard synergy assay. The activity of AR-12 against intracellular M. abscessus residing within macrophage was also evaluated. Finally, the potency of AR-12 *in vivo* was determined in a neutropenic mouse model that mimics pulmonary M. abscessus infection. AR-12 exhibited high anti-M. abscessus activity *in vitro*, with an MIC_50_ of 4 mg/liter (8.7 μM) and an MIC_90_ of 8 mg/liter (17.4 μM) for both subsp. *abscessus* and subsp. *massiliense*. AR-12 and amikacin exhibited comparable bactericidal activity against extracellular M. abscessus in culture. AR-12, however, exhibited significantly greater intracellular antibacterial activity than amikacin and caused a significant reduction in the bacterial load in the lungs of neutropenic mice infected with M. abscessus. No antagonism between AR-12 and clarithromycin, amikacin, imipenem, cefoxitin, or tigecycline was evident. In conclusion, AR-12 is active against M. abscessus *in vitro* and *in vivo* and does not antagonize the most frequently used anti-M. abscessus drugs. As such, AR-12 is a potential candidate to include in novel strategies to treat M. abscessus infections.

## Abstract (precis)

Therapeutic options for Mycobacterium abscessus infections are extremely limited. New or repurposed drugs are needed. The anti-M. abscessus activity of AR-12 (OSU-03012), reported to express broad-spectrum antimicrobial effects, was investigated *in vitro* and *in vivo*. Antimicrobial susceptibility testing was performed on 194 clinical isolates. Minimum bactericidal concentration and time-kill kinetics assays were conducted to distinguish the bactericidal versus bacteriostatic activity of AR-12.

## INTRODUCTION

Nontuberculous mycobacteria (NTM) are rapidly emerging opportunistic pathogens capable of causing severe lung infections in vulnerable individuals. Most countries report a dramatic increase in lung infections involving NTM in recent years (1). Among NTM species, Mycobacterium abscessus is one of the most challenging etiological agents (2). Infections caused by M. abscessus exhibit high rates of morbidity and mortality. Unfortunately, M. abscessus is typically resistant to most chemotherapies; treatment options are limited (3). Current, accepted therapeutic approaches require treatment with multidrug combinations over a long period of time (6 to 12 months). Regardless, treatment remains far from satisfactory (4–7). Furthermore, M. abscessus resistance to most antibiotics has begun to appear (8, 9). New or repurposed drugs are urgently needed.

AR-12 (OSU-03012), a novel celecoxib derivative that lacks cyclooxygenase-2 inhibitor activity, exerts a biological effect by a variety of mechanisms that include repressing host cell chaperone machinery, inhibiting kinase pathways, and upregulating autophagy (10–16). AR-12 was initially a small-molecule targeted anticancer agent administered orally (17–19). Recent studies indicate that AR-12 exerts a broad range of biological effects against a variety of microbial pathogens that includes fungi, bacteria, parasites, and viruses (10, 16, 20–27). Notably, AR-12 exhibits significant antimicrobial activity against intracellular bacteria *in vitro* and *in vivo* and is effective in eliminating certain multidrug-resistant (MDR) bacterial pathogens (12–14, 28–30). M. abscessus is a typical, MDR, facultative intracellular bacterium; the sensitivity of M. abscessus to AR-12 remains to be determined.

A detailed evaluation of the anti-M. abscessus activity of AR-12 was undertaken. AR-12 was active against M. abscessus growing *in vitro* and *in vivo* and proved especially effective in inhibiting the growth of intracellular organisms. Moreover, AR-12 did not act antagonistically toward the most frequently used anti-M. abscessus drugs. To our knowledge, this is the first report of the anti-M. abscessus activity of AR-12. As such, this work may provide a novel approach to treating M. abscessus infections.

## RESULTS

### AR-12 is active against *M. abscessus*.

A total of 194 clinical M. abscessus isolates (148 subsp. *abscessus* and 46 subsp. *massiliense*) were collected. AR-12 displayed similar antimicrobial activity against both subspecies. The MICs ranged from 2 to 16 mg/liter, with an MIC_50_ and an MIC_90_ of 4 and 8 mg/liter (8.7 and 17.4 μM), respectively, emphasizing the broad sensitivity of M. abscessus to AR-12 (Table 1; see also Table S1 in the supplemental material).

**TABLE 1:** MICs of AR-12 for 194 clinical M. abscessus isolates

M. abscessus subspecies	MIC range (mg/liter)	MIC_50_ (mg/liter)	MIC_90_ (mg/liter)	No. (%) of isolates sensitive to the indicated AR-12 concn			
				2 mg/liter	4 mg/liter	8 mg/liter	16 mg/liter
*abscessus* (*n* = 148)	2–16	4	8	9 (6.1)	72 (48.6)	64 (43.2)	3 (2.0)
*massiliense* (*n* = 46)	2–8	4	8	2 (4.3)	27 (58.7)	17 (37.0)	0 (0.0)

### AR-12 exhibits bactericidal activity.

The MBC/MIC ratio of AR-12 for all isolates tested ranged from 2 to >4 (Table 2). Most isolates (81.8%, 9/11) exhibited an MBC/MIC ratio of ≤4, which is indicative of bactericidal activity. AR-12 exhibited essentially the same bactericidal activity toward both subspecies. Indeed, no obvious trait (e.g., increased sensitivity to other antibiotics such as amikacin) differentiated the isolates that were and were not subject to the bactericidal activity of AR-12.

**TABLE 2:** MBC values and antibacterial activities of AR-12 against M. abscessus complex

Isolate	Subspecies	Concn (mg/liter)		MBC/MIC ratio	Antibacterial activity
		MIC	MBC^*a*^		
G74	*massiliense*	4	>16	>4	Bacteriostatic
G203	*massiliense*	4	16	4	Bactericidal
G124	*massiliense*	2	8	4	Bactericidal
G137	*massiliense*	4	8	2	Bactericidal
G141	*massiliense*	4	16	4	Bactericidal
G142	*abscessus*	4	16	4	Bactericidal
G164	*abscessus*	4	>16	>4	Bacteriostatic
G90	*abscessus*	4	16	4	Bactericidal
A311	*abscessus*	4	16	4	Bactericidal
A8	*abscessus*	4	8	2	Bactericidal
ATCC 19977	*abscessus*	4	16	4	Bactericidal

a A value of >16 mg/liter indicates an MBC value greater than the highest AR-12 concentration tested.

### AR-12 is comparable to amikacin in bacterial killing kinetics assay.

**FIG 1:**
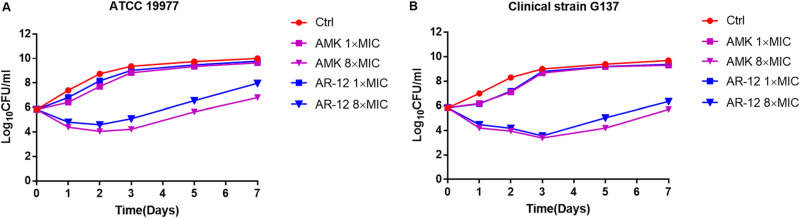
AR-12 and amikacin (AMK) exhibit comparable capacities to inhibit the extracellular growth of M. abscessus *in vitro*. (A) subsp. *abscessus* reference strain, ATCC 19977; (B) subsp. *massiliense* clinical isolate, G137.

To determine the potency of the killing kinetics of AR-12 against M. abscessus, AR-12 was tested at 1× and 8× MIC for 7 days; amikacin served as a positive control. At 1× MIC, there was no reduction in log_10_ CFU of either ATCC 19977 or G137 compared to the untreated, negative control (Fig. 1). Treatment with 1× MIC amikacin yielded comparable results. In contrast, treatment of subsp. *abscessus* and subsp. *massiliense* with 8× MIC of AR-12 resulted in ca. 2- and 3.3-log_10_ reductions in CFU, respectively, relative to the untreated control at 7 days. Amikacin exhibited comparable, concentration-dependent antimicrobial activity. Regrowth occurred after 3 to 7 days of incubation with either AR-12 or amikacin. Prior exposure to 1 mg/liter (0.25 MIC_50_), AR12 did not affect the MIC calculated subsequently for 16 clinical isolates or the M. abscessus subsp. *abscessus* reference strain (ATCC 19977) (unpublished data). Thus, it is unlikely that the regrowth shown here is due to increased antibiotic resistance. Rather, regrowth is more likely due to the loss of antibiotic potency with time in culture.

### AR-12 is indifferent in its interaction with five clinically important anti-*M. abscessus* drugs.

Indifference (fractional inhibitory concentration index [FICI] = 0.625 to 1.5) was the most commonly observed interaction (97.14%, 34/35) of all the antimicrobial combinations of AR-12 and the most frequently used anti-M. abscessus drugs (Table 3; Tables S2 and S3). Only the interaction of AR-12 with imipenem in a subsp. *abscessus* strain showed synergy (FICI = 0.5). No antagonism between drugs was observed.

**TABLE 3:** Indifferent interaction of AR-12 with antibiotics frequently used to treat M. abscessus infections^*a*^

Isolate	Subspecies	FICI^*b*^				
		AR-12 + CLA	AR-12 + AMK	AR-12 + IPM	AR-12 + CFX	AR-12 + TGC
G188	*massiliense*	0.75	1	0.75	1	1.25
A63	*massiliense*	1	0.75	1	1	1
G189	*massiliense*	1.5	0.625	0.75	0.75	1.25
G197	*abscessus*	1	1	0.5	1	1
G198	*abscessus*	1.25	1	0.625	0.75	1
A350	*abscessus*	0.75	1	0.75	1	1.5
ATCC 19977	*abscessus*	0.75	0.75	0.75	1	1

a Synergy between AR-12 and the five antibiotics was assessed using the broth microdilution checkerboard titration technique.

b Abbreviations: FICI, fractional inhibitory concentration index; CLA, clarithromycin; AMK, amikacin; IPM, imipenem; CFX, cefoxitin; TGC, tigecycline. FICI values: synergy (FICI ≤ 0.5), indifference (0.5 < FICI ≤ 4.0), and antagonism (FICI > 4.0).

### AR-12 inhibits the growth of intracellular *M. abscessus*.

**FIG 2:**
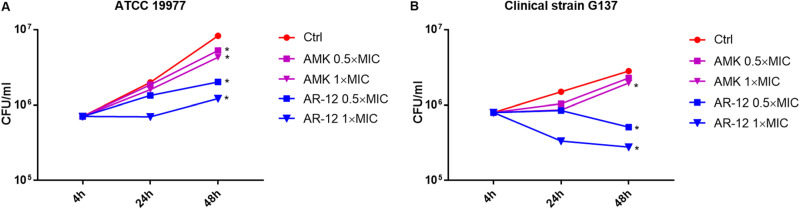
Relative intracellular antimicrobial activities of AR-12 and amikacin (AMK) expressed *in vitro*. (A) Subsp. *abscessus* reference strain ATCC 19977; (B) subsp. *massiliense* clinical isolate G137. *, *P* < 0.05 compared to control group.

Experiments were undertaken to access and compare the effects of AR-12 and amikacin on the intracellular survival of M. abscessus subsp. *abscessus* (reference strain ATCC 19977) and subsp. *massiliense* (clinical isolate G137 chosen at random) in primary peritoneal macrophages. Both AR-12 and amikacin at 0.5× and 1.0× MIC significantly inhibited the intracellular growth of subsp. *abscessus* relative to the untreated control; AR-12 demonstrated greater inhibition than amikacin (Fig. 2A). AR-12 exerted a far more pronounced effect on subsp. *massiliense*, which exhibited an ∼0.5 log_10_ reduction in intracellular organisms during 48 h incubation with 1× MIC (Fig. 2B). Treatment with 1× MIC amikacin, on the other hand, only slowed the intracellular growth M. abscessus subsp. *massiliense*.

### AR-12 inhibits the replication of *M. abscessus* in a mouse model of lung infection.

**FIG 3:**
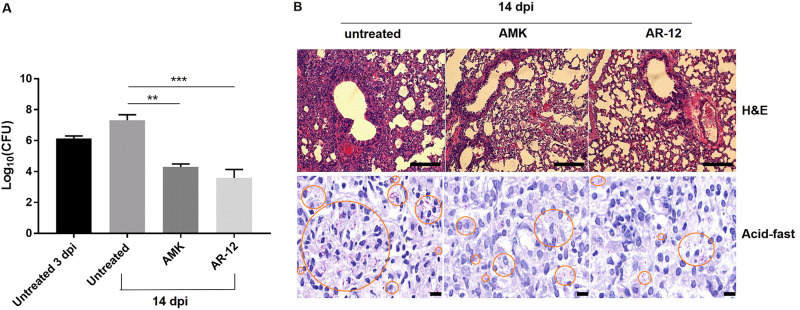
AR-12 exhibits anti-M. abscessus activity in a mouse lung infection model. (A) CFU assay, AMK, amikacin; dpi, days postinfection. The data are the mean CFU/lung tissues ± the standard deviations. Relative to the control untreated mice, lung tissues obtained from AMK- or AR-12-treated mice had significantly fewer organisms (**, *P* < 0.01; ***, *P* < 0.001). (B, top row) H&E staining. Scale bar, 100 μm. (B, bottom row) Acid-fast staining. Scale bar, 10 μm.

AR-12 exerted a potent inhibitory effect on the intra- and extracellular growth of M. abscessus *in vitro*. Additional experiments were conducted to evaluate and compare the effects of AR-12 and amikacin *in vivo*. A study was conducted using a murine respiratory infection model under immunocompromised conditions that mimicked the clinical presentations of NTM lung disease. AR-12 (50 mg/kg) and amikacin (50 mg/kg) caused significant reductions of **∼**3.7 log_10_ CFU and **∼**3.0 log_10_ CFU, respectively, in lungs accessed at 14 days postinfection (Fig. 3A). Immunostaining also revealed a reduction in the inflammatory pathology and bacterial counts in the lungs of AR-12-treated mice compared to untreated mice (Fig. 3B). Overall, AR-12 exhibited bactericidal activity *in vivo*, which was consistent its effects on the intracellular survival of M. abscessus *in vitro*.

## DISCUSSION

Treatment of M. abscessus infections is particularly difficult, a high rate of failure (45.1 to 46.0%) has been reported (6, 7). Developing new antimicrobial strategies to control M. abscessus is urgently needed. AR-12, used as a host-directed therapeutic or a direct antibacterial agent, is a prospective drug for treatment of multiple pathogens. It offers of variety of advantages such as oral delivery, a broad anti-pathogen spectrum, and effective in treating MDR bacteria (10–30). The efficacy of AR-12 in the treatment of M. abscessus infections remains to be determined.

In the present study, 194 clinical M. abscessus isolates were collected. AR-12 exhibited significant antimicrobial activity against these isolates *in vitro*; the MIC_50_ and MIC_90_ were 4 and 8 mg/liter (8.7 and 17.4 μM), respectively. Pharmacokinetic studies in mice indicated that the peak serum concentration of oral AR-12 administered at 200 mg/kg exceeded 20 μM. At the end of a 28-day period, the mice exhibited no visible signs of toxicity. All the animals maintained stable body weights and lacked gross pathological abnormalities upon necropsy (C.-S. Chen, unpublished data) (18). Similarly, an AR-12 dose of ≤200 mg/kg proved to be safe in mouse models of viral and fungal infections (22, 24).

Data obtained in a phase I clinical trial, exploring the effectivity of AR-12 as a targeted anticancer drug, showed that an 8 μM or 3.7 mg/liter serum concentration was safely attained and well tolerated by patients administered 800 mg twice daily (19). This serum concentration is similar to the MIC_50_ of AR-12 for M. abscessus *in vitro* determined here, indicating that a safe and effective dose can be achieved to treat clinical M. abscessus infections. The best dose and formulation of AR-12 required to optimize therapeutic outcomes, however, remain to be determined.

It has been suggested that AR-12 is a host-directed therapeutic rather than an antibiotic. Reportedly, it enhances host-dependent eradication of numerous intracellular infectious agents via multiple mechanisms that culminate in an antiproliferative effect (10–16). AR-12, for example, has no direct effect on *Francisella* strains but inhibits the intracellular survival of *Francisella* indirectly by inducing host cell autophagy (13). In a second study, however, AR-12 inhibited ampicillin- and kanamycin-resistant Escherichia coli and MDR N. gonorrhoeae directly by reducing DnaK expression (31). The latter study indicates that AR-12 can target certain bacteria and is a valuable, bioavailable small molecule that should be evaluated for the breadth of its antimicrobial activity. In the present study, AR-12 killed M. abscessus by a direct, concentration-dependent mechanism and was comparable to amikacin in activity expressed *in vitro*.

AR-12 was effective in inhibiting the growth of intracellular M. abscessus *in vitro* at concentrations effective in inhibiting organisms growing extracellularly. Similarly, AR-12 inhibited M. abscessus growth in a mouse model of lung infection. Interestingly, AR-12 exhibited a slight advantage over amikacin in these latter two cases, which was not evident when accessing the effects of AR-12 on extracellular growth *in vitro*. This finding was most likely due to the combined effects of the direct bactericidal activity of AR-12 and its host-directed therapeutic function. This is a significant advantage of AR-12 over other drugs used in treating bacteria, such as M. abscessus, growing intracellularly. It exhibits single-use bactericidal activity and simultaneously enhances bacterial clearance, acting as a small molecule adjuvant.

Hydrophobicity is a major concern associated with AR-12, making it difficult to achieve therapeutic doses. Modification and enhanced delivery systems may be needed to generate higher drug concentrations *in vivo* (11). In this regard, AR-12 encapsulated in acetalated dextran microparticles maintained pharmaceutical activity while exhibiting reduced cytotoxicity (27–29).

M. abscessus infections generally require treatment with multidrug combinations. AR-12 is an attractive option for treating M. abscessus infections, although its optimal effectiveness may necessitate its incorporation into multidrug therapy. In the present study, no antagonism was observed between AR-12 and five antibiotics (clarithromycin, amikacin, imipenem, cefoxitin, and tigecycline) found to be clinically important in treating M. abscessus infections according to guidelines (4, 5). Although there are currently no reports of AR-12 antagonism, one study showed that AR-12 can potentiate the antibacterial activity of polymyxins purportedly by modifying lipopolysaccharide, altering the bacterial outer membrane, and promoting polymyxin uptake (30). Koselny et al. reported that AR-12 could enhance the activity of fluconazole in a mouse model of cryptococcosis (25). AR-12 also acts as a potentiator in colistin (polymyxin E)-sensitive strains (32). These results indicate that AR-12 does not antagonize other drugs and suggest the ease with which AR-12 could be integrated into current anti-M. abscessus drug combinations.

This study is limited by the fact that all isolates used were obtained from a single hospital, Shanghai Pulmonary Hospital, a designated treatment center for Chinese tuberculosis and NTM infections, which attracts NTM cases from across the country. Acquisition of M. abscessus from other sources is difficult. Consequently, many of the isolates may be related genetically and/or epidemiologically. In conclusion, AR-12 is active against M. abscessus both *in vitro* and *in vivo* and does not antagonize other antibiotics most frequently used to treat M. abscessus infections. As such, AR-12 is a potential candidate to include in novel therapeutic anti-M. abscessus regimens.

## MATERIALS AND METHODS

### Bacterial strains.

A total of 194 clinical M. abscessus isolates were acquired retrospectively from sputum and bronchoalveolar lavage fluid specimens that were obtained between January 2014 and December 2017 and stored at Shanghai Pulmonary Hospital. Shanghai Pulmonary Hospital is one of the designated treatment centers for Chinese tuberculosis and NTM infections, attracting NTM cases from across the country. Both MGIT960 medium culture and *p*-nitrobenzoic acid testing were used as preliminary screening methods for NTM. The positive isolates were divided into subsp. *abscessus* and subsp. *massiliense* based upon multilocus sequencing of the *rpoB* and *erm*(41) genes. Staphylococcus aureus ATCC 29213, Mycobacterium peregrinum (ATCC 700686), and M. abscessus ATCC 19977 reference strains were purchased from the American Type Culture Collection (ATCC; Manassas, VA). M. abscessus was grown at 37°C on Middlebrook 7H10 (M7H10) agar plates supplemented with 10% oleic acid-albumin-dextrose-catalase (OADC) and 0.2% glycerol or with continuous shaking in Middlebrook 7H9 (M7H9) broth supplemented with 10% OADC and 0.05% Tween 80 (7H9sB). Suspensions of all isolates were stored at –80°C and cultured fresh before each assay. Middlebrook 7H9 broth, M7H10 agar, cation-adjusted Mueller-Hinton II broth (CAMHB), and OADC supplement were purchased from Becton, Dickinson, and Company (BD; Franklin Lakes, NJ).

### Antimicrobial agents.

AR-12 (OSU-03012) was purchased from MedChemExpress (Monmouth Junction, NJ). Clarithromycin, amikacin, imipenem, cefoxitin, and tigecycline were purchased from Sigma-Aldrich (St. Louis, MO). Stock solutions of AR-12 (128 μg/ml) were dissolved in 32% DMSO (4 μg/ml in 1%). Clarithromycin (2,048 μg/ml) was prepared in 100% DMSO, and other antibiotics at 2,048 μg/ml were prepared in deionized water. All antibiotics were stored as aliquots at –20°C and serially diluted just prior to experimental use.

### Antimicrobial susceptibility testing.

Antimicrobial susceptibility testing was conducted in accordance with CLSI document M24-A2. In brief, individual M. abscessus colonies were picked from M7H10 agar plates, grown to logarithmic phase in M7H9 broth, diluted to McFarland 0.5 with sterile saline, and adjusted to 1 × 10^5^ to 5 × 10^5^ CFU/ml using CAMHB. AR-12 was serially diluted 1:2 in a 96-well microtiter plate (final concentrations ranged from 16 to 0.008 μg/ml), and 100 μl of bacteria in suspended CAMH was added to each well. The plates were incubated at 37°C under aerobic conditions for 3 to 4 days until the control wells without AR-12 exhibited visible growth. Mycobacterium peregrinum (ATCC 700686) and Staphylococcus aureus (ATCC 29213) served as control reference strains. The MIC was defined as the lowest drug concentration at which no bacterial growth was observed by visual examination. All assays were repeated twice.

### Minimum bactericidal concentration.

The minimum bactericidal concentration (MBC) was determined in order to distinguish between the bacteriostatic and bactericidal activities of AR-12. M. abscessus subsp. *abscessus* and subsp. *massiliense* isolates were selected randomly for determination. After the antimicrobial susceptibility test at day 4 of AR-12 incubation, wells in which the drug concentrations were higher than the MIC were evenly resuspended. Portions (100 μl) of the contents from wells were serially diluted (10-fold) and plated to count the remaining CFU. The CFU were determined after 5 days of incubation at 37°C. The MBC values were defined as the minimum drug concentration with no colony growth. An antibiotic is considered bactericidal when the MBC/MIC ratio is ≤4; otherwise, it is considered bacteriostatic.

### Time-kill kinetics assay.

We used the subsp. *abscessus* ATCC 19977 and a clinical subsp. *massiliense* isolate (G137) to evaluate the potency of the killing kinetics of AR-12 against M. abscessus. Tubes (5 ml) of 7H9sB containing AR-12 or amikacin at 1× or 8× MIC were inoculated with 5 × 10^5^ CFU of bacteria growing exponentially; the tubes were incubated for 7 days at 37°C with shaking (100 rpm). The bacteria were enumerated on days 0, 1, 2, 3, 5, and 7 by plating serial dilutions on lysogeny broth agar plates; CFU were enumerated after an additional 5 days incubation at 37°C. All assays were performed in duplicate.

### Synergy with frequently used antimycobacterial drugs.

Synergy between AR-12 and clarithromycin, amikacin, imipenem, cefoxitin, and tigecycline was assessed *in vitro* using the broth microdilution chequerboard titration technique. Briefly, serial 2-fold dilutions of AR-12 were added to the columns of wells, and serial dilutions of the five antibiotics were added to the rows of wells in a 96-well microtiter plate, yielding a two-dimensional matrix composed of AR-12 in combination with various antibiotic concentrations. The 2-fold dilutions of AR-12 or each antibiotic alone and the medium without AR-12 or antibiotics were used as controls. Randomly selected M. abscessus subsp. *abscessus* and subsp. *massiliense* isolates (∼10^6^ CFU/ml) were added to each well, and the plates were incubated for 3 to 4 days at 37°C. The final MIC of each drug combination was determined as described for antimicrobial susceptibility testing above. The results of synergy testing were interpreted based on the FICI determined according to the following formula: FICI = (MIC of antibiotic A in combination/MIC of antibiotic A alone) + (MIC of AR-12 in the combination/MIC of AR-12 alone). Drug interactions exhibit three patterns: synergy (FICI ≤ 0.5), indifference (0.5 < FICI ≤ 4.0), and antagonism (FICI > 4.0).

### Intracellular killing.

Mouse peritoneal macrophages, frequently used in studies undertaken to examine the factors that affect intracellular replication of *Mycobacterium* species, were obtained by previously described methods (33–35). The cells were infected at a multiplicity of infection of 5 with M. abscessus ATCC 19977 suspended in RPMI 1640 with 10% fetal bovine serum and no antibiotics. After 4 h of incubation at 37°C in 5% CO_2_, the cells were washed three times with warm phosphate-buffered saline (PBS) to remove the extracellular organisms. Fresh RPMI 1640 supplemented with 10% human serum and then AR-12 or amikacin at 0.5× to 1× MIC was added; infected control cells were treated with culture medium alone. The cells were lysed with 0.05% sodium dodecyl sulfate at various times postinfection, and the numbers of CFU were quantified by plating serial dilutions of lysates on M7H10 agar plates. Cell viability was evaluated by trypan blue exclusion before and after infection and/or drug treatment at each time point. Peritoneal macrophages were 95% viable at 48 h postinfection; drug exposure did not affect cell viability (data not shown).

### Animal experiments.

Studies were conducted using an immunocompromised mouse model modified to mimic respiratory infection (36). Briefly, 24 6-week-old male BALB/c mice (20 to 22 g) were rendered neutropenic by intraperitoneal injection of cyclophosphamide at 150 mg/kg administered 4 days and 1 days prior to infection. The mice were then infected intranasally with ∼4 × 10^7^ CFU of M. abscessus ATCC 19977. On day 3 postinfection, three mice were sacrificed, and the CFU/lung were determined to evaluate the model’s efficacy. The remaining 21 infected mice were divided randomly into three groups: group 1 (control administered PBS with 0.5% methylcellulose–0.1% Tween 80 by daily oral gavage for 2 weeks), group 2 (treated with 50 mg/kg AR-12 in PBS with 0.5% methylcellulose–0.1% Tween 80 administered by daily oral gavage for 2 weeks), and group 3 (administered 50 mg/kg amikacin intramuscularly for 2 weeks daily). The dosage of amikacin was selected based upon the report of Das et al. (36). The same AR-12 dose was chosen to facilitate a comparison of antibacterial effects. The mice were sacrificed on day 17 postinfection, and the upper lobes of the right lungs of mice in each group were dissected, fixed with 4% paraformaldehyde for 24 h, and sectioned. The tissue sections were subjected to hematoxylin and eosin (H&E) and acid-fast staining, and the bacterial loads were compared by histologic examination. The remaining portions of the lungs were homogenized, and 10-fold serial dilutions of the homogenates were plated on M7H10 agar supplemented with 10% Middlebrook OADC enrichment. The bacterial content of the lungs was estimated from the colonies that grew on plates incubated for 5 days at 37°C. The animal experiments were approved by Tongji University School of Medicine review boards and performed according to established safety protocols in a biosafety level 2 laboratory at the Shanghai Public Health Clinical Center.

### Statistical analysis.

Statistical differences between study groups were determined by a nonpaired Student *t* test; a *P* value of <0.05 was considered significant. Computations were performed using Prism 7 (GraphPad Software, San Diego, CA).

## Supplementary Material

Supplemental file 1
