# Supplementary material for: AR-12 Exhibits Direct and Host-Targeted Antibacterial Activity toward Mycobacterium abscessus
Source: Antimicrob Agents Chemother. 2020 Jul 22;64(8):e00236-20. doi: 10.1128/AAC.00236-20 (PMC7526805; doi:10.1128/AAC.00236-20)
Supplement: Supplemental file 1 [file AAC.00236-20-s0001.pdf]

Supplementary Table 1. MIC of AR-12 for 194 *M. abscessus* isolates

**Isolate**	**Subspecies**	**MIC (mg/L)**
A8	*abscessus*	4
A10	*abscessus*	8
A25	*abscessus*	4
A35	*abscessus*	2
A38	*abscessus*	4
A39	*massiliense*	4
A40	*abscessus*	8
A49	*abscessus*	4
A51	*abscessus*	4
A54	*abscessus*	4
A58	*abscessus*	2
A59	*abscessus*	2
A63	*massiliense*	4
A69	*abscessus*	4
A73	*abscessus*	4
A79	*abscessus*	4
A126	*abscessus*	8
A137	*abscessus*	2
A173	*massiliense*	8
A175	*abscessus*	4
A176	*abscessus*	16
A182	*abscessus*	4
A183	*abscessus*	4

A186	*massiliense*	4
A189	*abscessus*	8
A197	*abscessus*	2
A205	*massiliense*	4
A213	*abscessus*	4
A215	*abscessus*	4
A217	*abscessus*	2
A218	*abscessus*	8
A222	*massiliense*	4
A228	*massiliense*	8
A232	*abscessus*	4
A233	*abscessus*	4
A243	*abscessus*	4
A244	*abscessus*	8
A247	*massiliense*	4
A249	*abscessus*	4
A254	*massiliense*	4
A255	*abscessus*	4
A266	*abscessus*	4
A267	*massiliense*	4
A268	*massiliense*	4
A274	*abscessus*	4
A289	*massiliense*	4
A295	*massiliense*	2
A297	*abscessus*	8

A305	*abscessus*	4
A311	*abscessus*	4
A312	*abscessus*	4
A315	*abscessus*	16
A317	*abscessus*	4
A321	*abscessus*	8
A323	*massiliense*	8
A329	*abscessus*	4
A330	*abscessus*	4
A337	*abscessus*	2
A350	*abscessus*	8
A353	*abscessus*	8
129	*abscessus*	8
G70	*abscessus*	4
3	*abscessus*	4
G72	*abscessus*	4
G73	*abscessus*	8
G74	*massiliense*	4
G75	*massiliense*	4
G76	*abscessus*	4
G77	*massiliense*	8
G78	*abscessus*	4
G79	*abscessus*	4
2	*abscessus*	4
G82	*abscessus*	8

G84	*abscessus*	8
G85	*massiliense*	4
G86	*abscessus*	8
G87	*massiliense*	8
G88	*massiliense*	4
G89	*abscessus*	8
G90	*abscessus*	4
G91	*abscessus*	8
G93	*abscessus*	4
G94	*abscessus*	4
G95	*massiliense*	8
G98	*massiliense*	4
G99	*abscessus*	8
289	*abscessus*	8
G101	*massiliense*	4
G102	*abscessus*	4
G103	*abscessus*	8
G104	*abscessus*	8
G105	*massiliense*	4
G106	*abscessus*	4
G107	*massiliense*	8
G108	*massiliense*	4
G109	*abscessus*	4
G110	*massiliense*	8
G111	*abscessus*	4

G112	*abscessus*	8
G113	*abscessus*	4
G114	*abscessus*	8
G115	*abscessus*	8
G116	*massiliense*	4
G117	*abscessus*	4
G118	*abscessus*	8
G119	*abscessus*	8
G120	*abscessus*	8
G121	*abscessus*	8
G122	*abscessus*	4
G123	*abscessus*	4
G124	*massiliense*	2
G125	*abscessus*	8
G126	*massiliense*	4
G127	*abscessus*	4
G128	*abscessus*	8
G129	*abscessus*	4
G132	*abscessus*	4
G133	*abscessus*	4
G134	*abscessus*	4
G135	*massiliense*	8
G136	*abscessus*	8
G137	*massiliense*	4
G138	*massiliense*	8

G139	*abscessus*	4
G140	*abscessus*	8
G141	*massiliense*	4
G142	*abscessus*	4
G143	*abscessus*	4
G144	*abscessus*	8
G145	*abscessus*	2
G146	*abscessus*	8
G147	*massiliense*	4
G148	*abscessus*	2
G149	*abscessus*	4
G150	*abscessus*	8
G151	*abscessus*	8
G152	*abscessus*	8
G153	*massiliense*	8
G155	*abscessus*	4
G156	*massiliense*	8
G157	*massiliense*	8
G158	*abscessus*	8
G159	*abscessus*	4
G160	*abscessus*	4
G161	*abscessus*	8
G162	*abscessus*	4
G163	*abscessus*	8
G164	*abscessus*	4

G165	*abscessus*	8
G169	*abscessus*	4
G170	*abscessus*	8
G172	*abscessus*	8
G173	*massiliense*	8
G174	*abscessus*	8
G175	*abscessus*	8
G176	*abscessus*	4
G177	*abscessus*	8
G178	*abscessus*	8
G179	*abscessus*	8
G180	*abscessus*	16
G181	*abscessus*	4
G182	*abscessus*	8
G183	*abscessus*	4
G184	*abscessus*	8
G185	*abscessus*	8
G186	*abscessus*	4
G187	*abscessus*	8
G188	*massiliense*	4
G189	*massiliense*	8
G190	*massiliense*	8
G192	*abscessus*	4
G193	*abscessus*	8
G194	*abscessus*	4

G195	*abscessus*	8
G196	*abscessus*	8
G197	*abscessus*	4
G198	*abscessus*	8
G199	*massiliense*	4
G200	*abscessus*	4
G201	*abscessus*	8
G203	*massiliense*	4
G204	*abscessus*	8
G205	*abscessus*	4
G206	*abscessus*	8
G207	*massiliense*	4
G208	*abscessus*	8
G210	*abscessus*	8
G211	*abscessus*	8
G213	*abscessus*	4
G215	*abscessus*	8
G216	*abscessus*	4
G218	*abscessus*	8
G219	*abscessus*	4
G220	*massiliense*	8

Supplementary Table 2. MIC values of AR-12 and five antibiotics effective in treating *M. abscessus*[a]

Isolate	subspecies	MIC of the drug indicated (mg/L)					
		AR-12	CLA	AMK	IPM	CFX	TGC
G188	*massiliense*	4	64	4	16	32	4
A63	*massiliense*	4	0.25	16	8	16	2
G189	*massiliense*	8	0.06	4	16	32	4
G197	*abscessus*	4	0.5	4	32	32	1
G198	*abscessus*	8	0.06	2	32	32	2
A350	*abscessus*	8	1	16	16	32	0.5
ATCC 19977	*abscessus*	4	0.5	8	8	16	2

[a]CLA, clarithromycin; AMK, amikacin; IPM, imipenem; CFX, cefoxitin; TGC, tigecycline; FICI, fractional inhibitory concentration index.

Supplementary Table 3. Indifferent interaction between AR-12 and five antimycobacterial drugs[a]

AR-12 + CLA			AR-12 + AMK			AR-12 + IPM			AR-12 + CFX			AR-12 + TGC		
MIC in combination (mg/L)		FICI[b]	MIC in combination (mg/L)		FICI	MIC in combination (mg/L)		FICI	MIC in combination (mg/L)		FICI	MIC in combination (mg/L)		FICI
AR-12	CLA		AR-12	AMI		AR-12	IPM		AR-12	CFX		AR-12	TGC	
1	32	0.75	2	2	1	1	8	0.75	2	16	1	1	8	1.25
2	0.125	1	2	4	0.75	2	4	1	2	8	1	2	1	1
4	0.06	1.5	4	0.5	0.625	2	8	0.75	4	8	0.75	2	4	1.25
2	0.25	1	2	2	1	1	8	0.5	2	16	1	2	0.5	1
2	0.06	1.25	4	1	1	4	4	0.625	4	8	0.75	4	1	1
2	0.5	0.75	4	8	1	4	4	0.75	4	16	1	4	0.5	1.5
1	0.25	0.75	2	2	0.75	1	4	0.75	2	8	1	2	1	1

[a]CLA, clarithromycin; AMK, amikacin; IPM, imipenem; CFX, cefoxitin; TGC, tigecycline; FICI, fractional inhibitory concentration index

[b]FICI, fractional inhibitory concentration index, which was determined by the following formula: FICI = (MIC of drug A in the combination/MIC of drug A alone) + (MIC of drug B in the combination/MIC of drug B alone). Drug interactions have three patterns: synergism (FICI≤0.5), indifference (0.5＜FICI≤4) or antagonism (FICI＞4.0).
